# Investigating genomic diversity of *Staphylococcus aureus* associated with pediatric atopic dermatitis in South Africa

**DOI:** 10.3389/fmicb.2024.1422902

**Published:** 2024-08-19

**Authors:** Gillian O. N. Ndhlovu, Kiran G. Javkar, Takudzwa Matuvhunye, Froodia Ngondoh, Dorota Jamrozy, Stephen Bentley, Adebayo O. Shittu, Felix S. Dube

**Affiliations:** ^1^Department of Molecular and Cell Biology, Faculty of Science, University of Cape Town, Cape Town, South Africa; ^2^Institute of Infectious Disease & Molecular Medicine, University of Cape Town, Cape Town, South Africa; ^3^Department of Computer Science, University of Maryland, College Park, MD, United States; ^4^Joint Institute for Food Safety and Applied Nutrition, University of Maryland, College Park, MD, United States; ^5^Parasites and Microbes Programme, Wellcome Sanger Institute, Hinxton, United Kingdom; ^6^Department of Microbiology, Obafemi Awolowo University, Ile-Ife, Osun, Nigeria; ^7^Institute of Medical Microbiology, University Hospital Munster, Munster, Germany

**Keywords:** genomic diversity 5, *Staphylococcus aureus*, pediatric, atopic dermatitis, South Africa

## Abstract

**Importance:**

*Staphylococcus aureus* frequently colonizes the skin and nose of patients with atopic dermatitis (AD), a disease associated with skin barrier dysfunction and chronic cutaneous inflammation. Published genomic studies on AD-associated *S. aureus* in pediatric populations in sub-Saharan Africa are limited.

**Objectives:**

To investigate the phenotypic and genomic diversity of *S. aureus* in children with and without AD during early childhood.

**Data, setting and participants:**

A cross-sectional study of 220 children (aged 9–38 months) with AD (cases) and without AD (controls) from Cape Town and Umtata, South Africa.

**Main outcomes and measures:**

*S. aureus* phenotypic and genomic diversity were investigated using whole-genome sequencing, antibiotic susceptibility testing and biofilm microtiter assay.

**Results:**

Of the 124 *S. aureus* isolates recovered from 220 children, 96 isolates (79 cases and 17 controls) with high-quality sequences were analyzed. Isolates from cases showed greater phenotypic resistance to gentamicin (10%), rifampicin (4%), chloramphenicol (4%), and exhibited multidrug resistance (9%) than in controls. Furthermore, the isolates from cases formed stronger biofilms than those from controls (76% vs. 35%, *p* = 0.001), but showed no dominance of any virulence factor gene or mobile genetic elements. There was no significant difference in the distribution of immune evasion cluster types between cases and controls. However, IEC type G was identified only among cases.

**Conclusion and relevance:**

AD-associated *S. aureus* has phenotypic and genetic features that are important for successful pathogenic colonization and survival. Further studies are needed to assess the pathological implications of colonization of various *S. aureus* lineages *in vivo* to elucidate their pathological contribution to AD pathogenesis and pathophysiology.

## Introduction

Pathological skin and nasal colonization with *Staphylococcus aureus* is common in patients with atopic dermatitis (AD)—a chronic or recurrent inflammatory skin disease which affects about 20% of children globally (Gilaberte et al., 2020). Due to the frequent association of *S. aureus* infections with AD, antibiotics are routinely used in the management of AD. However, the effectiveness of antibiotics in AD is limited by the rapid recolonisation with *S. aureus* following treatment cessation (di Domenico et al., 2019a). The excessive use of antibiotics may lead to significant changes in the skin microbial community and emergence of antibiotic-resistant isolates, which are associated with severe forms of AD (Jung et al., 2015; Shi et al., 2016). *S. aureus* contributes to AD pathology through virulence factors and biofilms, which are commonly detected in skin lesions of children with AD (Allen et al., 2014; Sonesson et al., 2017; di Domenico et al., 2018). Further, adhesins, toxins, proteases and significant changes in antigens enable the bacterium to adhere to host tissues, leading to impaired skin barrier function and inflammation (di Domenico et al., 2019b). Previous studies have shown that patients that are colonized with *S. aureus* strains which form strong biofilms, experience severe forms of the disease (Ogonowska et al., 2020; Gonzalez et al., 2021).

Some genes associated with antibiotic resistance, biofilm formation, and virulence in *S. aureus* are carried on mobile genetic elements (MGEs), such as plasmids, bacteriophages (prophages) and staphylococcal pathogenicity islands (SaPIs) (Malachowa and DeLeo, 2010). These are central to the adaptation of *S. aureus* in different environments (Malachowa and DeLeo, 2010). (Harkins et al., 2018) Moreover, lineage-specificity has been observed among MGEs (McCarthy et al., 2012; McCarthy and Lindsay, 2012). Recent data suggest that AD is associated with specific *S. aureus* lineages (Harkins et al., 2018), with clonal complex (CC) 1 being positively associated with AD in European children, while CC30 being negatively associated with AD (Rojo et al., 2014; Harkins et al., 2018). These differences in *S. aureus* lineages appear to be accompanied by differences in the carriage of virulence factors and adaptability to AD (Hwang et al., 2021).

It is therefore important to identify genomic markers of *S. aureus* that are associated with AD pathology in children with AD from their non-AD counterparts. Moreover, there is limited data on *S. aureus* strain biology from sub-Saharan Africa. We therefore investigated the phenotypic and genomic factors important for *S. aureus* adaptation on the skin and in the anterior nares of children with AD compared with healthy pediatric participants.

## Materials and methods

### Collection of *Staphylococcus aureus* isolates

We used the *S. aureus* isolates collected from our previously published work (Ndhlovu et al., 2021). Briefly, samples were collected using sterile nylon-tipped flocked swabs (Cat. no. 516C; Copan Italia, Brescia, Italy) from the lesional and non-lesional skin and anterior nares of children (*n* = 220) with and without AD from Umtata and Cape Town, South Africa (HREC/REF: 451/2014). The swabs were placed into skim milk-tryptone-glucose-glycerol (STGG) and transported to the laboratory within 2 h of collection at 4°C and stored in the freezers (−80°C) for further analysis. A total of 124 *S. aureus* isolates were recovered after the swabs were inoculated onto Mannitol Salt Agar (MSA) (National Health Laboratory Services [NHLS], Green Point Media Laboratory Cape Town, South Africa), and incubated at 37°C for 48 h in aerobic conditions. The *S. aureus* colonies were confirmed by testing for mannitol fermentation and DNase production (Kateete et al., 2010). Detailed information on the isolate collection can be found in Ndhlovu et al. (2021).

### Assessment of biofilm formation of *Staphylococcus aureus* isolates

*S. aureus* isolates were assessed for biofilm formation *in vitro* using the crystal violet biofilm microtiter assay (MTA) as previously described (Stepanović et al., 2007; Gonzalez et al., 2021). Biofilm biomass OD readings were interpreted as follows: (OD ≤ OD negative control [OD_c_], non-producer; OD_c_ < OD < 2·OD_c_, weak producer; 2·OD_c_ < OD < 4·OD_c_, moderate producer; and OD > 4·OD_c_, strong producer) (Stepanović et al., 2007).

### Antibiotic susceptibility testing of *Staphylococcus aureus* isolates

Antibiotic susceptibility testing (AST) was conducted using the Kirby-Bauer disk diffusion method as previously described and resistance to cefoxitin used as a surrogate for methicillin resistance (Wang et al., 2005). *S. aureus* ATCC 25923 was used as the quality control. The D-test was used to distinguish constitutive and inducible resistance to the macrolide-lincosamide-streptogramin B (MLS_B_) group of antibiotics (Yao et al., 2019). The AST was interpreted according to 2021 EUCAST guidelines and breakpoints. Isolates that were resistant to at least three classes of antibiotics were classified as multidrug-resistant (MDR) (Magiorakos et al., 2012).

### DNA extraction, library preparation, and whole-genome sequencing

Total DNA extraction was performed using a heat lysis method as described previously in Ndhlovu *et al* (Ndhlovu et al., 2021), and quantified using the Biotium Accuclear Ultra-high sensitivity dsDNA Quantitative kit. Samples were cherry-picked to 200 ng/120 μL using the Tecan liquid handling platform and sheared to 450 bp using a Covaris LE220 instrument.

Post sheared samples (in 96 well plates) were purified using SPRISelect beads and rearrayed into a in 384 plate on the Hamilton STAR. Library construction (ER, A-tailing, and ligation) was performed using the ‘NEB Ultra II custom kit’ on an Agilent Bravo WS automation system. PCR setup was performed using KapaHiFi Hot start mix and 384 well UDI tag barcodes on the Agilent Bravo WS automation system. PCR conditions were as follows: initial incubation at 95°C for 5 min followed by five cycles of 98°C for 30 s, 65°C for 30 s and 72°C for 1 min, and a final incubation step at 72°C for 10 min. Post PCR plates were pooled using equal volumes and purified using Agencourt AMPure XP SPRI beads on the Hamilton STAR. Libraries were quantified using an Agilent Bioanalyser, normalized to 2.8 nM, and prepared for sequencing on a NovaSeq (Page et al., 2016).

### Genome assembly, MLST analysis, gene prediction and gene clustering

All bioinformatics tools listed herewith were used with their default parameter settings unless any custom parameter was explicitly stated. Pre-processed reads were *de novo* assembled using Velvet v1.2.10 (Zerbino, 2010). The species identity of each genome was confirmed using KmerFinder v3.2 (Hasman et al., 2014; Larsen et al., 2014). High-quality genomes with <150 contigs and N50 > 50,000 (Manara et al., 2018) were included in the subsequent analyses. *In silico* multi-locus sequence typing (MLST) and *spa* typing were conducted using the MLST tool v2.0 and spaTyper v1.0, respectively. MLST clonal complexes (CCs) were inferred from PubMLST.^1^ Virulence factors were detected using the VirulenceFinder v2.0 tool (Kleinheinz et al., 2014) and the Virulence Factors of Pathogenic Bacteria (VFPB 2022) database (Liu et al., 2019). Antibiotic resistance determinants were identified using the Resistance Gene Identifier v5.2.0; accessed via the Comprehensive Antibiotic Resistance Database (CARD) v4.0.2 (Alcock et al., 2020). and ResFinder v4.4.3 (Florensa et al., 2022). The staphylococcal cassette chromosome *mec* (SCC*mec*) types of *mecA*-positive MRSA isolates were determined by the SCC*mec*Finder v1.2 (Kaya et al., 2018). Identification of putative plasmids was performed by using the PlasmidFinder v2.1.1 (Carattoli and Hasman, 2020). The size and associated genes of plasmids identified by PlasmidFinder were obtained from NCBI (Boratyn et al., 2013). The PHAge Search Tool Enhanced Release (PHASTER) algorithm (Arndt et al., 2016) was used to identify prophage sequences from the *S. aureus* genomes. Insertion sequence (IS) elements were identified from Prokka v1.14.6 (Seemann, 2014) and annotated genomes using the ISfinder v2.0 (Siguier et al., 2006). Analyses were limited to replicon, prophage, and IS elements identified in 10 or more genomes.

### Phylogenetic tree construction and annotation

Single nucleotide polymorphism based phylogenetic analysis was conducted using the Reference sequence Alignment based Phylogeny builder (REALPHY) v1.13 (Bertels et al., 2014) with default settings. *Staphylococcus aureus* NCTC 8325 was used as a reference. The generated phylogenetic trees were visualised and annotated using the iTOL (Interactive Tree of Life) tool v6 (Letunic and Bork, 2021).

### Statistical analyses

Unless stated otherwise, all statistical analyses were performed in STATA SE 15.1 (StataCorp LP, Texas, USA). The two-sample *z*-test was applied to compare the proportions. Cohen’s kappa statistic was performed to assess the agreement between phenotypic and genotypic susceptibility and resistance to antibiotics. The κ coefficient was interpreted as no agreement (κ < 0–0.2), minimal agreement (κ = 0.21–0.39), weak agreement (κ = 0.4–0.59), moderate agreement (κ = 0.6–0.79), strong agreement (κ = 0.8–0.9), and almost perfect agreement (κ >0.9) (McHugh, 2012).

## Results

### Participant characteristics and *Staphylococcus aureus* population structure

**Figure 1:**
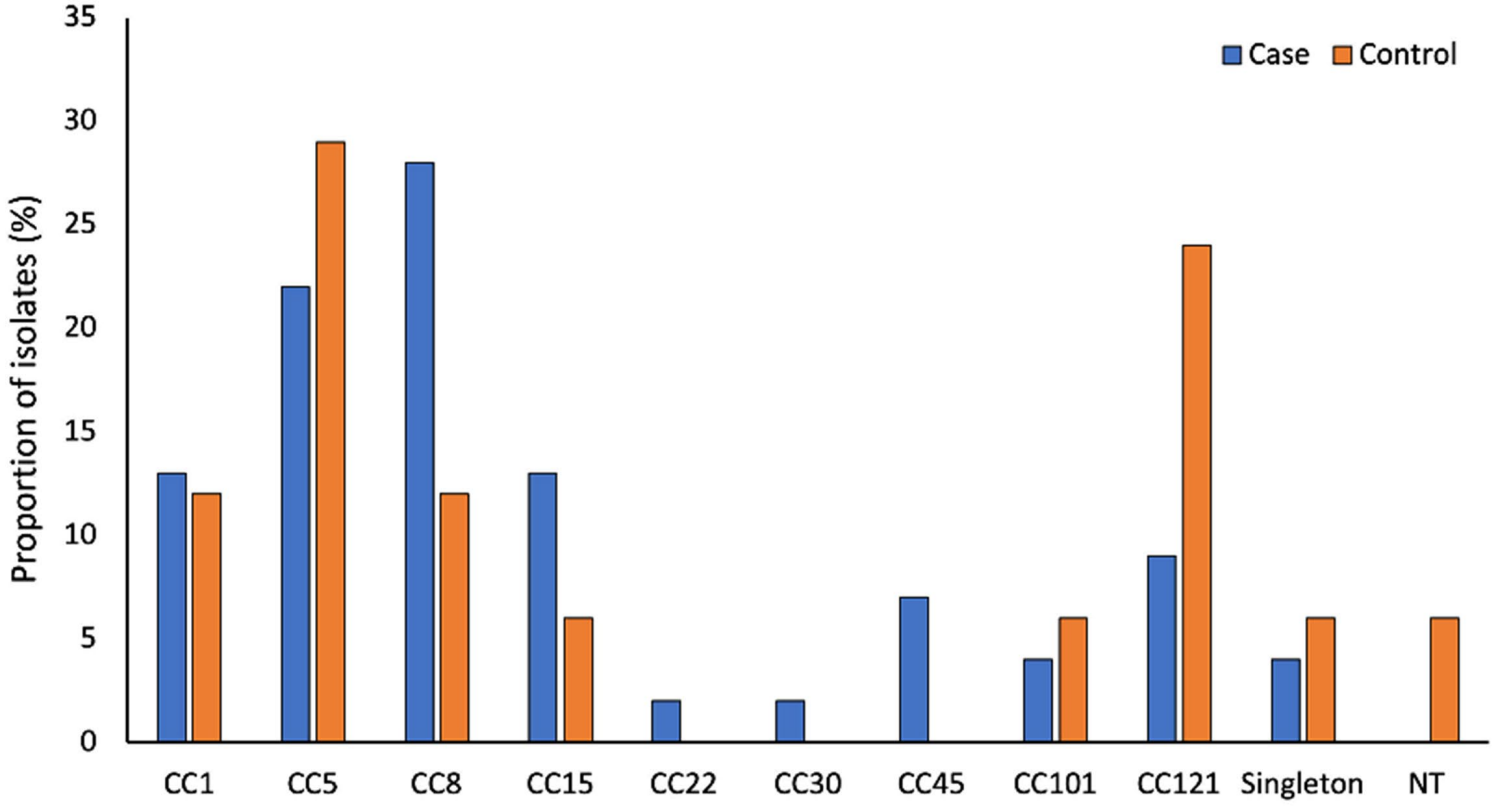
Genetic diversity of S. aureus associated with AD. CC1 (ST1), CC5 (ST5, ST4005, ST650), CC8 (ST8, ST72 and ST612), CC15 (ST15, ST199 and ST2126), CC22 (ST22), CC30 (ST31), CC45 (ST45 and SST508), CC101 (ST101) and CC121 (ST121). Singletons include ST12 (*n* = 2), ST20 (*n* = 1) and ST88 (*n* = 1).

Supplementary Table S1 describes the participant’s characteristics. Of the 124 *S. aureus* isolates, high-quality genomes were obtained from 96 isolates (79 cases and 17 controls) (see Materials and Methods section for the high-quality genome selection criteria). The average size of the assembled genomes was 2.80 Mb (2.71–2.98 Mb), with an average of 25 (10–79) contigs per genome. Each genome had approximately 2,665 (2538–2,936) predicted protein sequences and a GC content of 32.7% (32.6–32.9%). The *S. aureus* isolates were grouped into 20 STs, which further clustered into nine CCs and three singletons (Figure 1). Of these CC5 (ST5, ST650, and ST4005) and CC8 (ST8, ST72, and ST612) were predominant, accounting for 24% (23/96) and 25% (24/96) of the isolates, respectively (Supplementary Table S2).

### Distribution of mobile genetic elements (MGEs) among cases and controls

Plasmid analysis revealed 27 unique plasmids based on *rep* genes. Plasmid replicons were not identified in four isolates (4.2% [4/96]), all from cases. Lineage-association was limited to three plasmids, namely pBORa53 (CC8), SAP074A (CC5), and SAP057A (CC121) (Supplementary Table S3). Plasmids pN315, pSJH101 and pMSSA476, carrying penicillin (*blaZ*) and cadmium (*cadD*) resistance genes, were the most frequently identified in both cases (47% [37/79], 19% [17/79], and 41% [32/79], respectively) and controls (41% [7/17], 35% [6/17], and 24% [4/17], respectively) (Table 1). Plasmid pE-1(EDINA), which carries the enterotoxin E (*see*), *edinA*, as well as cadmium and arsenic resistance genes, was more common among controls than cases (29% [5/17] vs. 6% [5/79], *p* = 0.005) (Table 1).

**Table 1:** The distribution of plasmids, present in at least 10 genomes, in cases and controls.

Plasmid	Size	Associated genes	Cases (*n* [%])	Controls (*n* [%])	*p-*value
pN315	24,653 bp	*cadD, blaZ, blaR1, blaI, arsR, arsC, Tn552*	37 (47)	7 (41)	0.671
MSSA476	20,652 bp	*cadD, blaI, blaR1, blaZ*	32 (41)	4 (24)	0.1897
pSJH101	30,429 bp	*cadD, blaZ, blaR1*	17 (19)	6 (35)	0.2274
pS0385p1	5,246 bp	*tetB*	16 (20)	3 (18)	0.8067
pSaa6159	20,730 bp	*blaI, blaZ*	16 (20)	2 (12)	0.416
pBORa53	17,334 bp	*blaR1, blaZ, cadD, cadX*	11 (14)	2 (12)	0.8134
pDLK1	2,402 bp	*ermC*	10 (13)	1 (6)	0.4262
pSAS	20,652 bp	*cadD, cadC, blaR1, blaZ*	10 (13)	1 (6)	0.3497
pETB	38,211 bp	*etB, cadD*	7 (9)	3 (18)	0.282
pE-1(EDINA)	34,986 bp	*entE, edinA, cadX, cadD, arsR, Tn 431*	5 (6)	5 (29)	0.0047
pSK156	45,052 bp	*cadD, blaI, blaR1, blaZ, antP, qacA/B*	5 (6)	2 (12)	0.4342

We predicted 392 prophage genomes from 96 *S. aureus* isolates, of which, 150 (38%) were intact/complete. The 150 intact prophages were identified in 95% (91/96) of the isolates: 96% (76/79) of cases and 88% (15/17) of controls. All intact prophages belonged to the family *Siphoviridae* and various genera including *Biseptimavirus*, *Dubowvirus*, *Peeveelvirus*, *Phietavirus*, and *Triavirus* (Supplementary Table S4). Phage integrase genes were found in 68% (102/150) of the intact prophages. The predicted intact prophages were grouped into 31 distinct prophages. No significant association was observed between the lineage and the identified intact prophages (Supplementary Table S4). However, some prophages, such as *Staphylococcus* phages phiETA (CC121), SA97, and phiN315 (CC5), and phage 71 (CC45) only had one lineage (Supplementary Table S4). We limited our analysis to prophages that were identified in at least 10 genomes (Table 2). Of these, *Staphylococcus* prophage phi2958PVL was the most common (34% [33/96]) and was more frequently identified among cases than controls (41% [32/79] vs. 6% [1/17], *p* = 0.0064) (Table 2).

**Table 2:** The distribution of prophages, present in at least 10 genomes, in cases and controls.

Most similar prophage hit	Associated genes^†^	Case [*n* (%)]	Control [*n* (%)]	*p*-value
*Staphylococcus* prophage phi2958PVL	*lukF-PV*, *lukS-PV*, *vapE*, *clpP*	32 (41%)	1 (6%)	0.0064
*Staphylococcus* prophage phiJB	*yopX* of the type III secretion system	18 (23%)	3 (18%)	0.6420
*Staphylococcus* prophage P282	immune evasion cluster genes *chp*, *sak*, *scn* and *clpP*	16 (20%)	2 (12%)	0.416

^†^ The information of the associated genes was obtained from the NCBI database.

Table 3 shows the distribution of IS elements in cases and controls. ISSau3 was detected in all case and control isolates. ISSau5 was only identified in cases (14% [11/79]) and ISSep3 was more prevalent in cases than controls (54% [43/79] vs. 29% [5/17], *p* = 0.061). Also, ISCsp1 and ISSau6 were more common among controls than cases (71% [12/17] vs. 37% [29/79], *p* = 0.0104 and 53% [9/17] vs. 23% [18/79], *p* = 0.0121, respectively).

**Table 3:** The distribution of IS elements, present in at least 10 genomes, in cases and controls.

IS element	IS family	Species	Case (*n* [%])	Control (*n* [%])	*p*-value
ISSau3	IS1182	*Staphylococcus aureus*	79 (100)	17 (100)	–
IS1181	ISL3	*Staphylococcus aureus*	48 (61)	11 (65)	0.762
ISSep3	IS200/IS605	*Staphylococcus epidermidis*	43 (54)	5 (29)	0.061
IS1252	IS30	*Enterococcus* sp.	39 (49)	5 (29)	0.134
ISBli29	ISNCY	*Brevibacterium linens*	31 (39)	10 (59)	0.139
ISCsp1	IS3	*Clostridium* sp.	29 (37)	12 (71)	0.0104
ISSsu9	IS1595	*Streptococcus suis*	33 (42)	7 (41)	0.964
ISSau6	IS6	*Staphylococcus aureus*	18 (23)	9 (53)	0.0121
IS655	IS3	*Bacillus halodurans*	16 (20)	2 (12)	0.416
IS431R	IS6	*Staphylococcus aureus*	16 (20)	2 (12)	0.416
ISSau2	IS3	*Staphylococcus aureus*	10 (13)	4 (24)	0.249
ISSau8	ISL3	*Staphylococcus aureus*	8 (10)	4 (24)	0.1296
ISSau5	IS30	*Staphylococcus aureus*	11 (14)	0 (0)	–
ISSep1	IS1182	*Staphylococcus epidermidis*	10 (13)	1 (6)	0.426

### Prevalence of phenotypic and genotypic antibiotic non-susceptibility

Phenotypic susceptibility to all antibiotics was similar in cases (61%; 48/79) and controls (59%; 10/17, *p* = 0.882) (Supplementary Figure S2). Phenotypic resistance to chloramphenicol (4% [3/77]), gentamicin (9% [7/77]) and rifampicin (4% [3/78]) was low, being detected exclusively among cases. The prevalence of MRSA was also low in both cases (8% [6/78]) and controls (6% [1/17]). Phenotypic MDR isolates were only detected in cases (9% [7/79]) (Supplementary Figure S1). *In silico* determination of antibiotic resistance revealed ARGs for methicillin (*mecA*), tetracycline (*tet45, tetM*, and *tetK*), gentamicin (*acc*(*6′*)*-le-aph*(*2″*)*-la*), rifampicin (double mutation in RpoB; H_481_N and I_527_M), chloramphenicol (*cat*(*pC221*)), and MLS_B_ (*ermC* and *msrA*) (Supplementary Figure S3). There was moderate to almost perfect concordance between phenotypic and genotypic resistance to all antibiotics tested, except for trimethoprim-sulfamethoxazole (Supplementary Table S6). MRSA was identified in cases comprising the multidrug-resistant CC5-ST5-SCC*mec*IV and CC8-ST612-SCC*mec*IV (Supplementary Figures S5, S6).

### Phenotypic biofilm formation and biofilm-associated genes

Overall, most isolates were strong biofilm producers (72% [69/96]), followed by moderate (17% [16/96]) and weak (11% [11/96]). Strong biofilm producers were more common in cases (76% [60/79]) than in controls (35% [6/17], *p* = 0.001). The prevalence of isolates with moderate (14% [11/79] cases vs. 29% [5/17] controls, *p* = 0.120) and weak biofilm formation (10% [8/79] cases vs. 18% [3/17] controls, *p* = 0.377) was similar in the two groups (Figure S4).

### Prevalence of virulence genes

**Figure 2:**
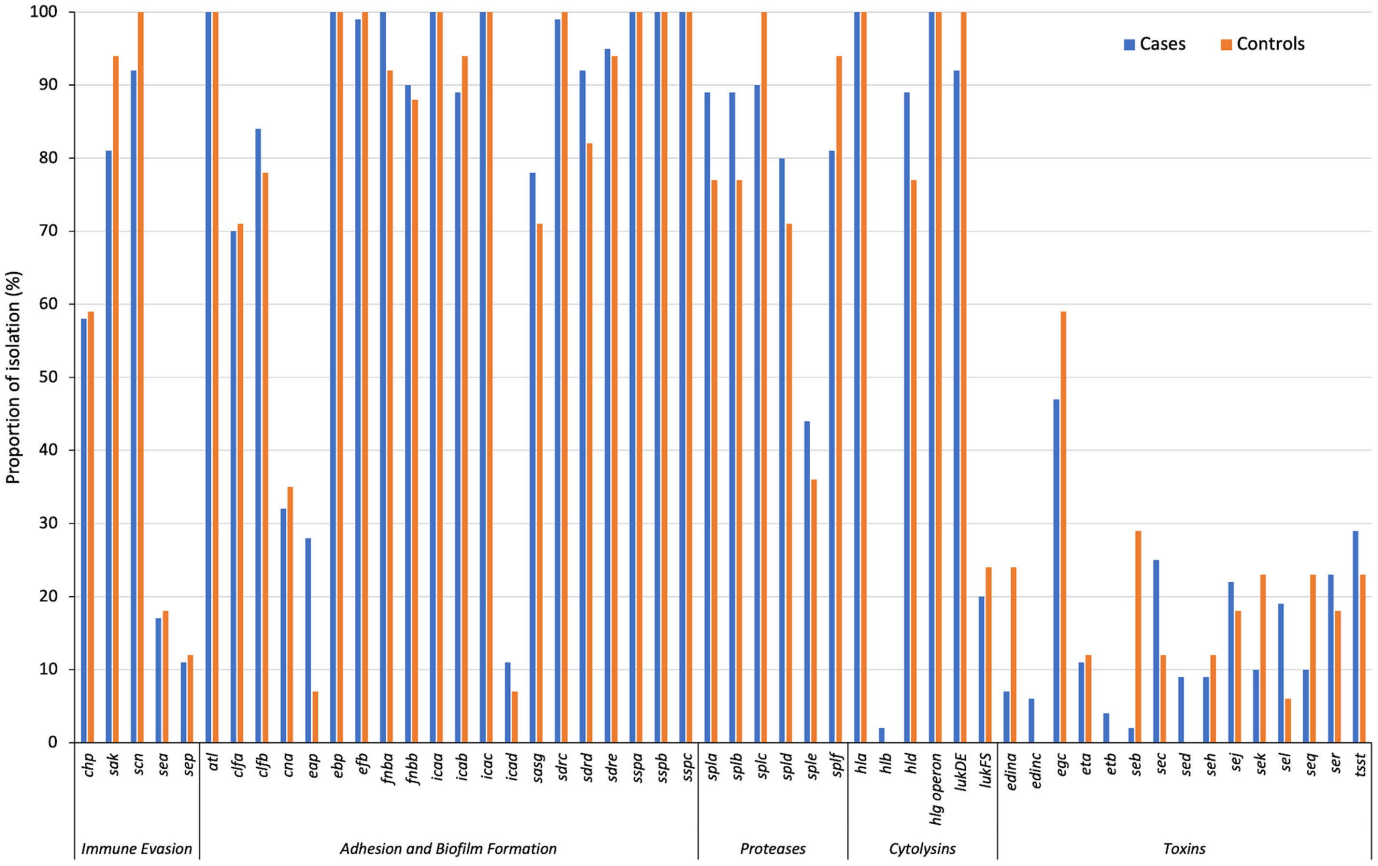
The distribution of virulence-associated genes in cases and controls, grouped by functional class. *p < 0.05; ***p < 0.001.

There was a low prevalence of staphylococcal superantigen genes, except for the enterotoxin gene cluster (EGC; comprises of *seg*, *sei*, *sem*, *sen*, *seo*, and *seu*) (48% [46/96]) (Figure 2). About 20% (20/96) of the isolates analysed were positive for the cytolysin genes *lukF-PV* and *lukS-PV*. More than 90% (90/96) of all isolates were *lukED*-positive. The toxin genes *edinC* (5% [4/79]), *etB* (4% [3/79]) and *sed* (9% [7/79]) were detected only in cases. Furthermore, *edinA* and *seb* were more frequently identified among controls than cases (24% [4/17] vs. 6% [5/79], *p* = 0.049 and 29% [5/17] vs. 1% [1/79], *p* = 0.001, respectively) (Figure 2).

Clustering of CCs with distinct antibiotic and virulence gene profiles Figure S6. CC1 isolates were *seh*-positive, while 58% (7/12) and 75% (9/12) were positive for the *lukFS-PV* and *sea/sek/seq* genes, respectively. The *egc* cluster was present in all CC5-ST5, CC8-ST72, CC121 and CC45-ST508 isolates. Most CC8-ST8 isolates carried *tsst* (78% [14/18]) and *sej* (72% [13/18]). The exfoliative toxin encoding genes (*eta* and *etb*) were detected only in CC15-ST15 (*n* = 4; *eta* only) and CC121 (n = 8; *eta* and or *etb*). The epidermal cell differentiation inhibitor (EDIN) genes were identified only in CC5-ST5 (*n* = 9, *edinA*) and CC121 (*n* = 4; *edinC*).

### Distribution of the immune evasion cluster genes

Regarding the immune evasion cluster (IEC) genes, most isolates carried *scn* (94%, 90/96), followed by *sak* (83%, 80/96), *chp* (57%, 55/96), *sea* (17%, 16/96) and *sep* (11% [11/96]); with frequencies similar between cases and controls (Figure S5). Six isolates, all from cases, did not carry any of the IEC genes. Of the various IEC groups, type B (*sak*-*chp*-*scn*) was predominant (37% [33/90]), followed by type E (*sak*-*scn*; 22% [20/90]), type D (*sea*-*sak*-*scn*; 13% [12/90]), type C (*chp*-*scn*; 11% [10/90]), type F (*sep*-*sak*-*chp*-*scn*; 8% [7/90]), and type A (*sea*-*sak*-*chp*-*scn*) and type G (*sep*-*sak*-*scn*); each with four isolates (4%).

## Discussion

This study shows that *S. aureus* isolates associated with AD tended to form stronger biofilms and carried genetic features that are linked to antibiotic resistance and virulence with potential clinical relevance to AD pathology.

The low prevalence of antibiotic resistance in our study is consistent with previous reports from Irish (Harkins et al., 2018), Polish (Wrobel et al., 2018), and Italian (di Domenico et al., 2018) pediatric patients with AD. The prevalence of MRSA strains was also low in both cases (8%) and controls (6%), as previously demonstrated (Harkins et al., 2018). Moreover, MDR *S. aureus* isolates were only detected among cases, suggesting frequent use of antibiotics or exposure to resistant strains, possibly from a hospital environment. Considering the low prevalence of AD in our setting, the proportion of MDR strains among children with AD may not reflect the true burden. Larger studies may be needed to further explore the impact of *S. aureus* antibiotic resistance on AD pathology.

*S. aureus* produces a variety of toxins, cytotoxins, proteases, antigens and adhesins that are key to its successful colonization, pathogenicity, and survival (di Domenico et al., 2019b). Epidemiological studies have shown that AD-associated *S. aureus* carry more virulence genes than controls (Na et al., 2012; Rojo et al., 2014; Cavalcante et al., 2015; Wang et al., 2016), which is also linked to increased disease severity (Rojo et al., 2014). Presently, the cases did not exhibit a predominance of virulence factor genes. Although no significant virulence factor genes were observed, it is likely that the disease environment could trigger more expression of the virulence factors resulting in disease pathology (Poh et al., 2022).

Biofilm formation is associated with increased disease severity (Pascolini et al., 2011; Allen et al., 2014; di Domenico et al., 2018). Particularly strong biofilms, protect *S. aureus* from (i) environmental factors (including antibiotics), (ii) the bactericidal effects of host antimicrobial peptides (AMPs) and, (iii) host immune responses (di Domenico et al., 2019a). In this regard, strong biofilm producers were more common among cases similar to previous studies (Allen et al., 2014; Sonesson et al., 2017; di Domenico et al., 2018). This suggests that AD-associated *S. aureus* isolates were better able to form biofilms and adapted for persistent colonization. These findings support the importance of strong biofilm formation in driving persistent colonization in children with AD. Also, persistent colonization with *S. aureus* in AD may be detrimental to the child as this provides a continuous source of virulence factors that will further perpetuate the clinical manifestations of AD and contribution to disease flares. As such, treatment strategies in AD need to have anti-biofilm capabilities to effectively reducing *S. aureus* colonization and mitigating its harmful effects.

Immune evasion by *S. aureus* is mediated by the immune evasion cluster (IEC), which modulates innate immune responses in humans (Verkaik et al., 2011). IEC includes SCIN, and a varying combination of SAK, CHIPS and staphylococcal enterotoxins, SEA or SEP, across different IEC types (van Wamel et al., 2006). We detected IEC genes in the *S. aureus* control group and most (92%) of the cases. Further, cases and controls did not differ substantially in the distribution of IEC types. Similar to previous reports (van Wamel et al., 2006; Verkaik et al., 2011; Silva et al., 2020), IEC type B, were independent of disease status, whereas the type G isolates were only identified among cases (Benito et al., 2016). Although the specific functions of the IEC genes and their contribution to AD are addressed in the literature (Rojo et al., 2014; Jian et al., 2021), the clinical relevance of different IEC types in AD is lacking and warrants further study.

Insertion sequences (IS) are small MGEs and are generally between 700–2,500 bp, affecting the genomic plasticity, diversity, and pathogenic potential of *S. aureus* (Kuroda et al., 2001). We found that ISCsp1 and ISSau6 were more common among controls. While there was no IS element more common in cases than controls, we observed that ISSau3, IS1181, ISSep3 were the three most frequently detected IS elements in cases. ISSau3 has been shown to increase resistance to beta-lactam antibiotics (Wang et al., 2021), the roles of IS1181 and ISSep3 are poorly studied in literature, especially in disease context.

There is increasing evidence that specific *S. aureus* lineages are adapted for induction of disease activity and survival in AD patients (Yeung et al., 2011; Byrd et al., 2017; Fleury et al., 2017; Harkins et al., 2018). In this study, we noted no significant difference in the clonality of *S. aureus* isolates from cases and controls. However, CC8 isolates predominated among cases, while CC121 isolates were the most common among controls. The specific features or mechanisms that favor the predominance of certain lineages in AD remain unknown (Geoghegan et al., 2018). However, it has been suggested that in AD, differences in the virulence potential across lineages are associated with the proliferation of some lineages and other lineages are associated with asymptomatic colonization in controls (Harkins et al., 2018).

Limitations to this study include the small sample size and our investigation focused on the recovery of a single colony which does not provide a comprehensive understanding of the clonality of *S. aureus* colonization in AD (Byrd et al., 2017). Lastly, we noted a higher prevalence of incomplete and questionable prophages compared to intact prophages. This is likely because intact prophages are usually under strong selection or genetic degradation for rapid deletion from bacterial genomes (Lawrence et al., 2001). However, since genomes are divided into contigs, some prophage sequences may have been split into different contigs and therefore misidentified as incomplete or questionable prophages by PHASTER (Marques et al., 2021). Moreover, the PHASTER tool also relies on previously annotated prophage sequences and, therefore, cannot identify novel prophage sequences. Subsequent studies will be focused on exploring the overall genomic factors beyond a specific set of genes, using tools like PRAWNS (Javkar et al., 2023).

## Conclusion

This study provides an understanding of the genomic differences of *S. aureus* in early childhood AD, in a previously unstudied population in South Africa. Although we did not observe a clear phylogenetic and clonal differentiation of *S. aureus* based on AD disease, specific phenotypic and genetic signatures distinguished AD isolates from controls. Specifically, AD-associated isolates exhibited phenotypic features related to biofilm formation, and genomic features associated with DNA damage repair and toxins which are important for the persistent colonization and propagation of AD disease.

## Data Availability

Supplementary results on *S. aureus* colonization based on AD and health, *S. aureus* colonization based on disease severity, study spa types and MLST sequence types are available in the in the Supplementary Data Sheet S3. The sequence data presented in this study are available on online repositories, European Nucleotide Archive (accession numbers are in Supplementary Table S2).

## Footnotes

^1^https://pubmlst.org/
