# Supplementary material for: Investigating genomic diversity of Staphylococcus aureus associated with pediatric atopic dermatitis in South Africa
Source: Front Microbiol. 2024 Aug 19;15:1422902. doi: 10.3389/fmicb.2024.1422902 (PMC11366657; doi:10.3389/fmicb.2024.1422902)
Supplement: Supplementary file 2 [file Data_Sheet_2.docx]

### **Supplementary Materials**

**Table S1.** Participant characteristics

|  | **Children with AD** | **Healthy children** |
| --- | --- | --- |
| No. of participants | 107 | 87 |
| Male, % (n/N) ^a^ | 56 (58/104) | 59 (51) |
| Rural, % (n) | 57 (61) | 61 (53) |
| Age in months, median (range) | 22.4 (9–38) | 22.1 (12–36) |
| Objective SCORAD | 41.5 (21.4–82.2) | NA |
| No. of *S. aureus* isolates | 104 | 20 |

^a^ Sex data is not available for three participants.

**Table S2.** Distribution of sequence types (ST) in cases and controls (ERS7013540)

| Sequence type | Cases, n (%) | Controls, n (%) | *p-*value |
| --- | --- | --- | --- |
| ST1 | 10 (12.6) | 2 (11.8) | 0.920 |
| ST101 | 3 (3.8) | 1 (5.9) | 0.696 |
| ST12 | 2 (2.5) | 0 (0) | 0.507 |
| ST121 | 7 (8.9) | 4 (23.5) | 0.085 |
| ST15 | 8 (10.1) | 1 (5.9) | 0.586 |
| ST199 | 1 (1.2) | 0 (0) | 0.641 |
| ST20 | 1 (1.2) | 0 (0) | 0.641 |
| ST2126 | 1 (1.2) | 0 (0) | 0.641 |
| `ST22 | 1 (1.2) | 0 (0) | 0.641 |
| ST31 | 1 (1.2) | 0 (0) | 0.641 |
| ST4005 | 3 (3.8) | 0 (0) | 0.414 |
| ST45 | 1 (1.2) | 0 (0) | 0.641 |
| ST5 | 13 (16.4) | 5 (29.4) | 0.214 |
| ST508 | 4 (5.1) | 0 (0) | 0.343 |
| ST612 | 2 (2.5) | 0 (0) | 0.507 |
| ST650 | 1 (1.2) | 0 (0) | 0.641 |
| ST72 | 4 (5.1) | 0 (0) | 0.343 |
| ST8 | 16 (20.2) | 2 (11.8) | 0.416 |
| ST88 | 0 (0) | 1 (5.9) | 0.030 |
| Unknown ST | 0 (0) | 1 (5.9) | 0.030 |

**Table S3.** Predicted plasmids.

| Plasmid | Species of origin | Size | No. of genomes | Accession no. | Associated CCs |
| --- | --- | --- | --- | --- | --- |
| pIM13 | *Bacillus subtilis* | 2246 bp | 1 | M13761 | CC8 (1) |
| pUSA01 | *Staphylococcus aureus* | 3125 bp | 2 | NC007790 | CC45 (2) |
| pETB | *Staphylococcus aureus* | 38211 bp | 10 | NC003265 | CC45 (3), CC121 (7) |
| pSJH101 | *Staphylococcus aureus* | 30429 bp | 23 | CP000737 | CC5 (8), CC8 (13), ST20 (1), NT (1) |
| pSAS | *Staphylococcus aureus* | 20652 bp | 11 | BX571858 | CC1 (6), CC8 (4), CC30 (1) |
| ORF1(EDINA) | *Staphylococcus aureus* | 34986 bp | 10 | AP003089 | CC5 (8), ST88 (1), NT (1) |
| pWBG759 | *Staphylococcus aureus* | 28384 bp | 23 | GQ900401 | CC5 (8), CC8 (13), ST20 (1), NT (1) |
| pNE131 | *Staphylococcus epidermidis* | 2355 bp | 1 | NC001390 | CC8 (1) |
| MSSA476 | *Staphylococcus aureus* | 20652 bp | 36 | BX571857 | CC1 (1), CC8 (28) |
| pBORa53 | *Staphylococcus aureus* | 18334 bp | 13 | AY917098 | CC8 (13) |
| pSK156 | *Staphylococcus aureus* | 45052 bp | 7 | GQ900448 | CC5 (7) |
| pSaa6159 | *Staphylococcus aureus* | 20730 bp | 18 | CP002115 | CC1 (1), CC8 (1), CC15 (11), CC45 (1), CC101 (4) |
| pTW20 | *Staphylococcus aureus* | 29585 bp | 8 | FN433597 | CC5 (6), CC8 (2) |
| pWBG745 | *Staphylococcus aureus* | 38204 bp | 2 | GQ900389 | CC1 (1), CC15 (1) |
| pWBG746 | *Staphylococcus aureus* | 33702 bp | 1 | GQ900390 | NT (1) |
| SAP057A | *Staphylococcus aureus* | 39308 bp | 4 | NC013334 | CC121 (4) |
| SAP074A | *Staphylococcus aureus* | 27268 bp | 8 | GQ900426 | CC5 (8) |
| SAP102A | *Staphylococcus aureus* | 36082 bp | 1 | GQ900496 | CC22 (1) |
| SAP105A | *Staphylococcus epidermidis* | 26236 bp | 1 | GQ900452 | CC5 (1) |
| pLW043 | *Staphylococcus aureus* | 57889 bp | 3 | AE017171 | CC8 (3) |
| repA(pWBG759) | *Staphylococcus aureus* | 28384 bp | 1 | GQ900401 | ST20 (1) |
| repC(Cassette) | *Staphylococcus aureus* | 68256 bp | 1 | AB037671 | ST88 (1) |
| repC(pS0385p1) | *Staphylococcus aureus* | 5246 bp | 19 | AM990993 | CC8 (18), ST88 (1) |
| repD(pK214) | *Lactococcus lactis* | 29871 bp | 1 | NC009751 | CC5 (1) |
| repD(pTZ4) | *Staphylococcus aureus* | 4555 bp | 3 | NC010111 | CC5 (3) |
| repL(pDLK1) | *Staphylococcus aureus* | 2402 bp | 11 | GU562624 | CC8 (6), CC121 (5) |
| repSAP001(pN315) | *Staphylococcus aureus* | 24653 bp | 44 | AP003139 | CC1 (7), CC5 (2), CC8 (16), CC15 (11), CC22 (1), CC30 (1), CC45 (1) CC101 (4), ST88 (1) |

Abbreviations: CC, clonal complex; ST, sequence type; NT, non-typeable (according to the MLST tool 2.0 in <https://www.genomicepidemiology.org/>).

Only predicted plasmids in bold (identified in ten or more genomes) were included in the main results and discussion.

**Table S4** Predicted intact prophages.

| **Most similar phage hit** | **No. of genomes** | **No. with integrase** | **Integrase group^†^** | **Predicted genus** | **Predicted family** | **Associated CCs** |
| --- | --- | --- | --- | --- | --- | --- |
| Staphylococcus phage P282 | 18 | 10 | Sa3int | Biseptimavirus | Siphoviridae | CC8 (16), CC45 (2) |
| Staphylococcus phage SA1014ruMSSAST7 | 2 | 0 |  | Biseptimavirus |  | ST12 (2) |
| Staphylococcus phage SA345ruMSSAST8 | 1 | 1 |  | Biseptimavirus |  | CC1 (1) |
| Staphylococcus phage phiN315 | 4 | 3 | Sa3int | Biseptimavirus |  | CC5 (4) |
| Staphylococcus phage phiSa2wa ST22 | 1 | 1 | Sa2int | Biseptimavirus |  | CC22 (1) |
| Staphylococcus phage 11 | 2 | 2 | Sa5int | Dubowvirus |  | CC5 (1), CC15 (1) |
| Staphylococcus phage 53 | 9 | 2 | Sa7int | Dubowvirus |  | CC5 (3), CC8 (6) |
| Staphylococcus phage SA97 | 5 | 3 | Sa1int | Dubowvirus |  | CC5 (5) |
| Staphylococcus phage phiETA2 | 1 | 1 | Sa2int | Dubowvirus |  | CC1 (1) |
| Staphylococcus phage phiNM2 | 1 | 1 | Sa7int | Dubowvirus |  | CC8 (1) |
| Staphylococcus phage AJ 2017 | 8 | 6 |  | Peeveelvirus |  | CC1 (6), CC121 (2) |
| Staphylococcus phage SA780ruMSSAST101 | 4 | 0 |  | Peeveelvirus |  | CC101 (4) |
| Staphylococcus phage SA7 | 3 | 2 |  | Peeveelvirus |  | CC1 (2), CC8 (1) |
| Staphylococcus phage tp310-1 | 1 | 1 | Sa2int | Peeveelvirus |  | CC5 (1) |
| Staphylococcus phage tp310-3 | 1 | 1 | Sa3int | Peeveelvirus |  | CC22 (1) |
| Staphylococcus phage 71 | 4 | 2 |  | Phietavirus |  | CC45 (4) |
| Staphylococcus phage 88 | 1 | 1 | Sa5int | Phietavirus |  | CC1 (1) |
| Staphylococcus phage 96 | 5 | 5 | Sa9int | Phietavirus |  | CC1 (4), CC15 (1) |
| Staphylococcus phage B236 | 1 | 0 | Sa1int | Phietavirus |  | CC8 (1) |
| Staphylococcus phage phiETA3 | 3 | 3 | Sa3int | Phietavirus |  | CC15 (3) |
| Staphylococcus phage phiETA | 8 | 7 | Sa1int | Phietavirus |  | CC121 (8) |
| Staphylococcus phage phiJB | 21 | 14 | Sa6int | Phietavirus |  | CC1 (3), CC5 (7), CC8 (4), CC15 (2), CC45 (1), ST12 (2), ST88 (1), NT (1) |
| Staphylococcus phage phiMR11 | 1 | 1 | Sa12int | Phietavirus |  | CC15 (1) |
| Staphylococcus phage 47 | 1 | 1 | Sa2int | Triavirus |  | CC1 (1) |
| Staphylococcus phage Ipla35 | 1 | 1 |  | Triavirus |  | CC121 (1) |
| Staphylococcus phage SA13 | 1 | 1 |  | Triavirus |  | CC5 (1) |
| Staphylococcus phage StauST398-2 | 2 | 2 |  | Triavirus |  | CC15 (1), CC121 (1) |
| Staphylococcus phage YMC/09/04/R1988 | 1 | 0 | Sa2int | Triavirus |  | CC5 (1) |
| Staphylococcus phage phi2958PVL | 33 | 30 | Sa2int | Triavirus |  | CC1 (9), CC5 (6), CC8 (6), CC15 (6), CC30 (1), CC45 (5) |
| Staphylococcus phage PT1028 | 5 | 0 | Not assigned | Unclassified |  | CC8 (2), CC121 (3) |
| Staphylococcus phage SPbeta like | 1 | 1 | Not assigned | Unclassified |  | CC8 (1) |

Abbreviations: CC, clonal complex; ST, sequence type; NT, non-typeable (according to the MLST tool 2.0 in <https://www.genomicepidemiology.org/>).

Only predicted prophages in bold (identified in ten or more genomes) were included in were included in the main results and discussion.

^†^Data on integrase groups obtained from Nepal *et al*. (2021)^61^, ^62^, and ^63^.

**Table S5.** Predicted insertion sequence elements.

| **IS element** | **IS family** | **Species of origin** | **No. of genomes** | **Length (bp)** | **Inverted repeat length (bp)^†^** | **ORF^§^** | **Accession no.** | **Associated CCs** |
| --- | --- | --- | --- | --- | --- | --- | --- | --- |
| IS1181 | ISL3 | *Staphylococcus aureus* | 59 | 1513 | 17/23 | 439 (120-1439) | L14544 | CC5 (22), CC8 (24), CC22 (1), CC121 (10), ST12 (1), NT (1) |
| IS1252 | IS30 | *Enterococcus* sp. | 44 | 1065 | 21/28 | 226 (75-755) | L38972 | CC8 (20), CC15 (11), CC22 (2), CC30 (1), CC45 (5), CC101 (4), ST88 (1) |
| IS232 | IS21 | *Bacillus thuringiensis* | 1 | 2184 | 48/67 | 431 (93-1388)  250 (1378-2130) | M38370 | CC22 (1) |
| IS256 | IS256 | *Staphylococcus aureus* | 3 | 1324 | 17/26 | 390 (102-1274) | M18086 | CC8 (2), CC22 (1) |
| IS257-1 | IS6 | *Staphylococcus aureus* | 3 | 791 | 21/26 | 224 (57-731) | X53952 | CC8 (3) |
| IS257-2 | IS6 | *Staphylococcus aureus* | 2 | 790 | 20/26 | 221 (57-722) | X53951 | CC8 (2) |
| IS257-3 | IS6 | *Staphylococcus aureus* | 3 | 789 | 23/27 | 224 (57-731) | X53951 | CC8 (3) |
| IS257R1 | IS6 | *Staphylococcus aureus* | 6 | 790 | 18/20 | 224 (57-731) | X13290 | CC8 (5), CC22 (1) |
| IS257R2 | IS6 | *Staphylococcus aureus* | 5 | 789 | 23/27 | 224 (57-731) | X13290 | CC8 (5) |
| IS431R | IS6 | *Staphylococcus aureus* | 18 | 790 | 17/20 | 224 (57-731) | M18437 | CC8 (18) |
| IS431mec | IS6 | *Staphylococcus aureus* | 7 | 790 | 18/20 | 224 (57-731) | X53818 | CC5 (1), CC8 (6) |
| IS655 | IS3 | *Bacillus halodurans* | 18 | 1221 | 9/15 | 94 (80-364)  288 (325-1191)  370 (80-1191) | NC_002570 | CC1 (12), CC30 (1), CC45 (5) |
| IS712 | IS21 | *Lactococcus lactis* | 1 | 2163 | 41/51 | 407 (81-1304)  252 (1316-2074) | NC_009004 | CC22 (1) |
| ISBli29 | ISNCY | *Brevibacterium linens* | 41 | 4278 | 32/38 | 197 (704-111)  119 (1060-701)  208 (1259-1885)  480 (1905-3347)  264 (3445-4239) | CP014869 | CC1 (11), CC5 (3), CC8 (2), CC15 (11), CC30 (1), CC45 (4), CC101 (4), CC121 (3), ST88 (1), NT (1) |
| ISClsp1 | IS3 | *Clostridium* sp. | 41 | 1222 | 25/41 | 96 (78-368)  291 (329-1204)  375 (78-1204) | NZ_AAQV01000022 | CC5 (22), CC22 (1), CC45 (1), CC101 (4), CC121 (11), ST88 (1), NT (1) |
| ISEfa8 | IS3 | *Enterococcus faecium* | 6 | 1366 | 30/41 | 96 (75-365)  305 (320-1237)  387 (75-1237 | NZ_AAAK03000001 | CC22 (1), CC45 (5) |
| ISLgar5 | IS256 | *Lactococus garvieae* | 2 | 1336 | 21/26 | 396 (95-1285) | AKFO01000017.1 | CC8 (2) |
| ISSau1 | IS30 | *Staphylococcus aureus* | 1 | 1070 | 20/26 | 315 (114-1061) | NC_002952 | CC30 (1) |
| ISSau2 | IS3 | *Staphylococcus aureus* | 14 | 1600 | 29/40 | 251 (34-789)  297 (705-1601)  521 (34-1601) | NC_002952 | CC22 (1), CC30 (1), CC121 (11), ST12 (1) |
| ISSau3 | IS1182 | *Staphylococcus aureus* | 96 | 1946 | 13/16 | 548 (280-1926) | NC_002952 | CC1 (12), CC5 (22), CC8 (24), CC15 (11), CC22 (1), CC30 (1), CC45 (5), CC101 (4), CC121 (11), ST12 (2), ST20 (1), ST88 (1), NT (1) |
| ISSau5 | IS30 | *Staphylococcus aureus* | 11 | 1136 | 7/12 | 163 (104-595)  175 (597-1124)  340 (104-1124) | NC_002952 | CC8 (8), CC15 (1), CC121 (1), ST20 (1) |
| ISSau6 | IS6 | *Staphylococcus aureus* | 27 | 793 | 21/22 | 224 (59-733) | NC_002952 | CC5 (13), CC8 (1), CC22 (1), CC45 (3), CC121 (6), ST20 (1), ST88 (1), NT (1) |
| ISSau8 | ISL3 | *Staphylococcus aureus* | 12 | 1498 | 19/24 | 438 (141-1457) | NC_007622 | CC1 (1), CC121 (11) |
| ISSep1 | IS1182 | *Staphylococcus epidermidis* | 11 | 1936 | 19/28 | 559 (271-1950) | NC_004461 | CC5 (1), CC8 (9), CC22 (1) |
| ISSep2 | IS110 | *Staphylococcus epidermidis* | 1 | 1564 |  | 337 (442-1455) | NC_004461 | ST20 (1) |
| ISSep3 | IS200/IS605 | *Staphylococcus epidermidis* | 48 | 740 |  | 160 (133-615) | NC_004461 | CC1 (12), CC8 (24), CC15 (11), CC22 (1) |
| ISSsu9 | IS1595 | *Streptococcus suis* | 40 | 1796 | 22/26 | 341 (89-1114)  157 (1217-1690) | NC_012926 | CC1 (1), CC5 (6), CC8 (17), CC15 (3), CC22 (1), CC101 (4), CC121 (8) |

Abbreviations: bp, base pair; IS, insertion element; ORF, open reading frame; CC, clonal complex; ST, sequence type; NT, non-typeable (according to the MLST tool 2.0 in <https://www.genomicepidemiology.org/>).

Only insertion sequences in bold (identified in ten or more genomes) were included in the main results and discussion.

^†^ left of slash is number of matching nucleotides, right of slash is total length of inverted repeat.

^§^ first number is amino acid length, range in parentheses is transcription start and end positions.

**Table S6.** Agreement between phenotypes and genotypes of antibiotic resistance.

| Antibiotic | Phenotypic resistance  (n [%]) | Genotypic resistance  (n [%]) | Agreement | Kappa | Comment on agreement | p-value |
| --- | --- | --- | --- | --- | --- | --- |
| Tetracycline | 20 (21%) | 20 (21%) | 91.67% | 0.7474 | Moderate | <0.0001 |
| Erythromycin | 14 (15%) | 11 (12%) | 94.79% | 0.7706 | Moderate | <0.0001 |
| Clindamycin | 11 (12%) | 8 (8%) | 96.88% | 0.8252 | Strong | <0.0001 |
| Cefoxitin | 7 (7%) | 6 (6%) | 98.96% | 0.9175 | Almost perfect | <0.0001 |
| Gentamicin | 7 (7%) | 6 (6%) | 98.96% | 0.9175 | Almost perfect | <0.0001 |
| Chloramphenicol | 4 (4%) | 4 (4%) | 100% | 1 | Almost perfect | <0.0001 |
| Trimethoprim-sulfamethoxazole | 4 (4%) | 45 (47%) | 56.1% | 0 | None | 0.5 |
| Rifampicin | 3 (3%) | 2 (2%) | 98.96% | 0.7949 | Moderate | <0.0001 |
| Mupirocin | 0 | 0 | — | — |  | — |

**Figure S1.** Bar plot representing the number of antibiotic classes to which *S. aureus* strains were phenotypically resistant. MDR, multidrug resistant. All comparisons between cases and controls were without statistical significance.


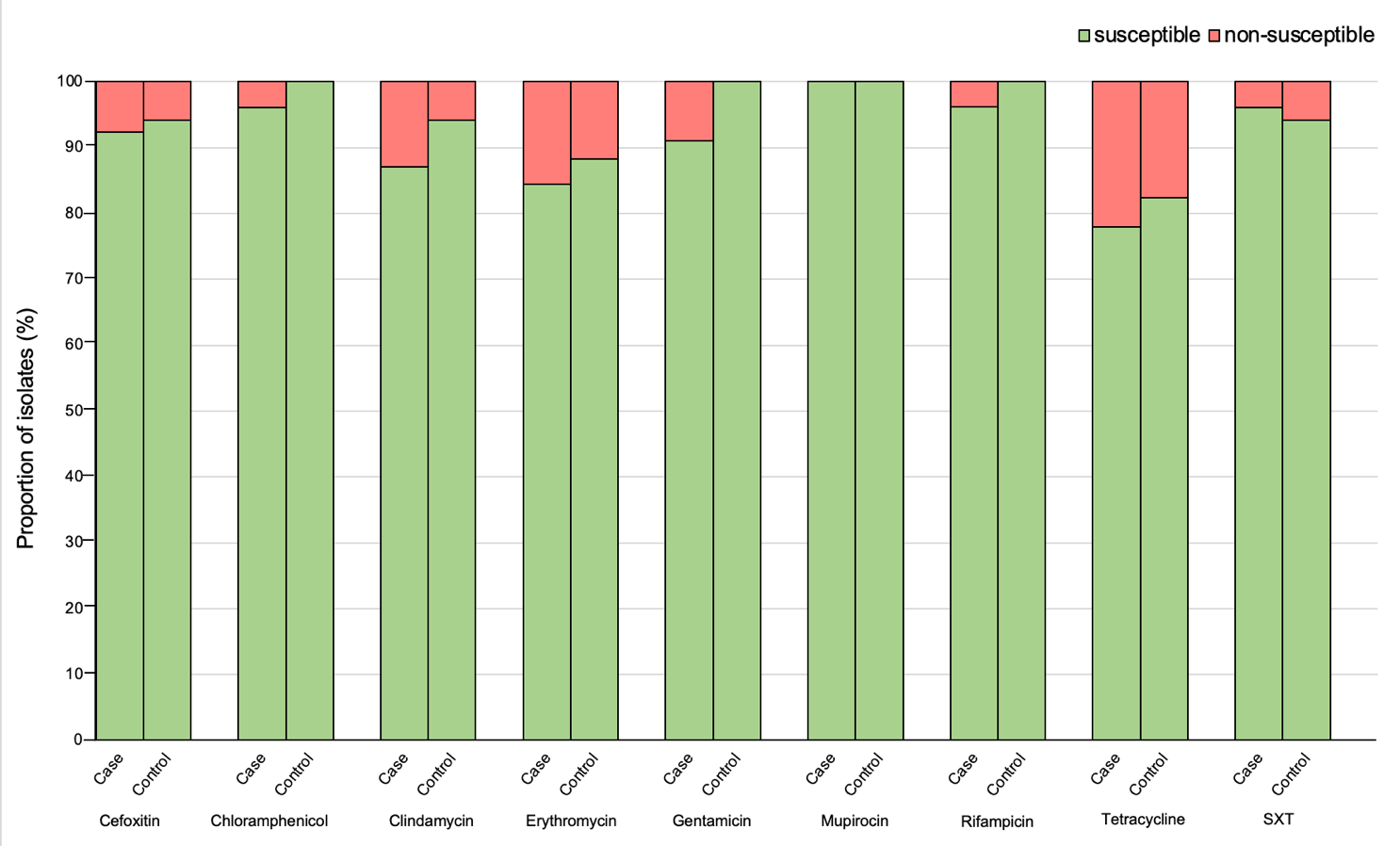


Figure S2. Phenotypic antibiotic resistance in cases and controls. SXT, trimethoprim-sulfamethoxazole.

**Figure S3.** The distribution of antimicrobial resistance genes (ARGs) in *S. aureus* from cases and controls. MLS_B_, macrolide-lincosamide-streptogramin B.

**Figure S4.** Distribution of biofilm phenotypes in cases and controls based on *S. aureus* lineages. Singletons include ST12 (n=2), ST20 (n=1) and ST88 (n=1).

Figure S5: The distribution of immune evasion cluster (IEC) types in cases and controls. IEC types A (sea-sak-chp-scn), B (sak-chp-scn), C (chp-scn), D (sea-sak-scn), E (sak-scn), F (sep-sak-chp-scn), and G (sep-sak-scn). All comparisons between cases and controls were not significant


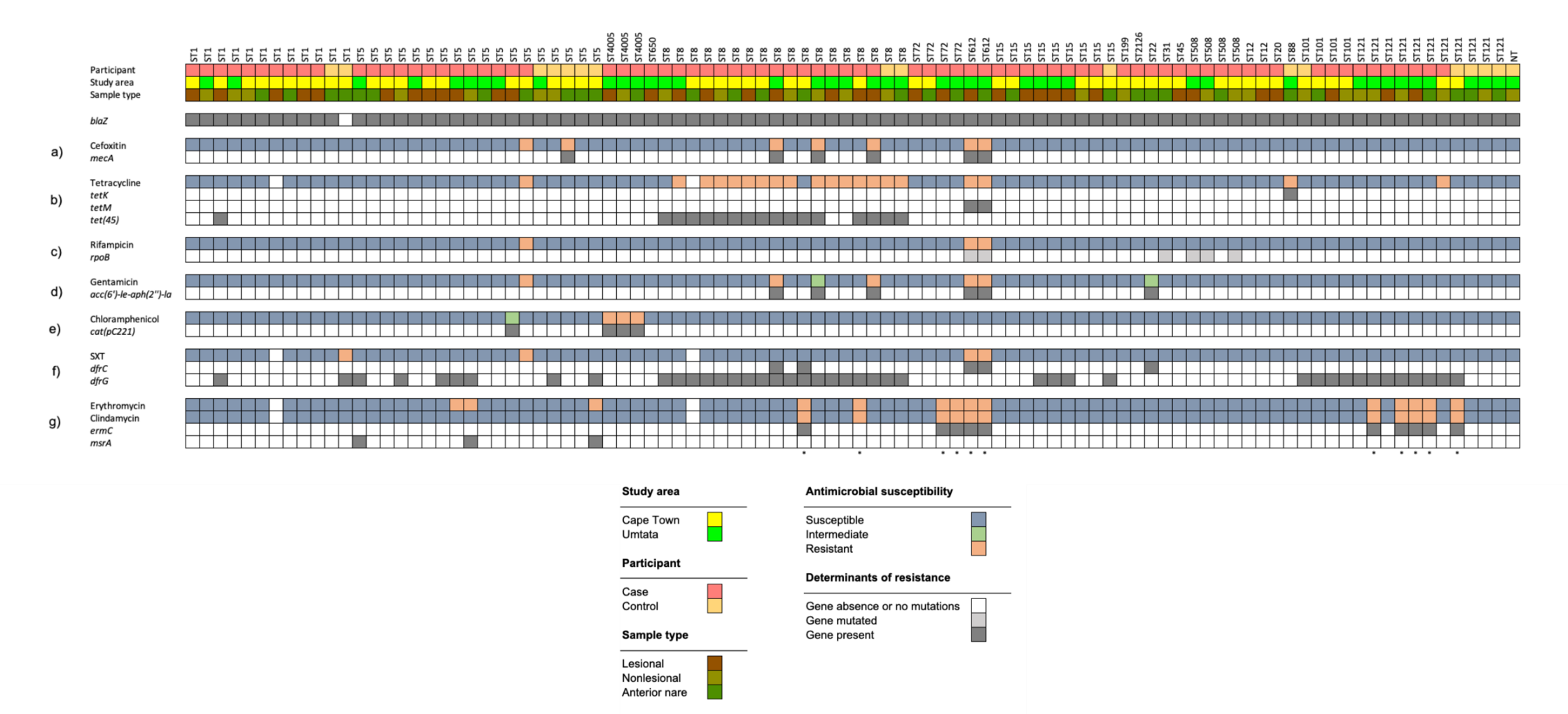


**Figure S6.** Resistome and phenotypic antibiotic susceptibility of *S. aureus* isolates based on *in silico* predicted sequence types. Asterix (*) under g) indicates isolates that exhibited phenotypic erythromycin-inducible clindamycin resistance based on the D-zone test. AST was not performed for the isolates with white boxes for tetracycline, rifampicin, gentamicin, chloramphenicol, erythromycin, and clindamycin due to lack of viability
